# The Nutrition and Dietetics Workforce Needs Skills and Expertise in the New York Metropolitan Area

**DOI:** 10.5539/gjhs.v8n6p14

**Published:** 2015-09-28

**Authors:** Ann Gaba, Apoorva Shrivastava, Chioma Amadi, Ashish Joshi

**Affiliations:** 1 CUNY School of Public Health, Hunter College, New York, USA; 2 CUNY School of Public Health, New York, USA

**Keywords:** nutrition, dietetics, education, employment, job skills

## Abstract

**Background:**

There is an increased demand in the Nutrition and Dietetics field which has fostered credentialing to ensure competent graduates. The objective of this study is to conduct an exploratory analysis to identify nutrition/dietetics workforce needs, skills and expertise in the New York metropolitan area as exemplified in position announcements over a 4 year period.

**Methods:**

We recorded position announcements for jobs in nutrition and dietetics from the New York State Registered Dietitian Yahoo group, and the Hunter College Nutrition and Food Sciences student and alumni listserv (NFS-L) over a 4 year period. Keywords were identified using job categories defined by the Academy of Nutrition and Dietetics (AND) compensation and benefits survey. This served as a starting point to enumerate the types of positions that have been posted for the New York metropolitan area in recent years.

**Results:**

Four hundred and twelve (412) unique job postings were recorded. Various educational levels, credentials, and skills desired by these employers were identified, assessed, and compared with similar data from the “supply side” reports from AND.

**Conclusions:**

The credentials and skills most desired by employers are similar to some of the learning objectives set forth for DPD and DI programs by ACEND, but not entirely congruent. The need for both client/customer focus and computer literacy may be implicit in the standards, but a more overt inclusion of these skills would likely be of benefit to ensure these are inculcated into every program and student.

## 1. Introduction

The field of Nutrition and Dietetics has recorded considerable growth over the past decade, largely attributed to the increasing awareness of the importance of nutrition in population health, and its crucial role in the management of a variety of chronic diseases, which constitute the leading causes of mortality and disability in the United States (Rogers, 2014; Kris-Etherton, Akabas, & Stemler, 2001). This has stimulated an increase in the demand for this discipline and fostered credentialing to ensure competent professionals. The Bureau of Labor Statistics has estimated a projected increase by 20% in the demand for employment of qualified nutritionists and dietitians over the next 5 years, exceeding other similar professions (Bureau of Labor Statistics, 2013). The growing market need for qualified dietitians and nutritionists continues to generate relevant concerns by key stakeholders which include:

1. The need for assuring competency of graduates in meeting essential work place requirements; a concern by the task force of the Academy of Nutrition and Dietetics, AND (Academy of Nutrition and Dietetics, 2012)
2. Uncertainty among prospective and current nutrition students regarding the existence of suitable employment opportunities in the field (Academy of Nutrition and Dietetics, 2012);
3. Inconsistency in employment conditions and expectations for dietitians and nutritionists in different geographical regions of the country (Rogers, 2014) and
4. The variability in qualifications required for the different dietitian/nutritionist positions advertised.

These concerns have created the need for strengthening the current and incoming dietetic workforce and improving and ensuring uniform standards in preparation and continuing education for employment opportunities in this field.

Dietitians who have received formal training and registration (RDs and DTRs) are collectively known as credentialed dietetic practitioners, and they constitute the professional dietetic workforce in the United States (Hooker, Williams, Papneja, Sen, & Hogan, 2012). These professionals must possess an associate’s degree (DTRs) or baccalaureate degree (RDs) in dietetics along with an accredited supervised practice program (Hooker et al., 2012). However, concern for the expected decline in the dietetic workforce in the coming decade as many older practitioners retire, and the ultimate need to strengthen preparation of dietetics and nutrition graduates to meet marketplace demands have led to the AND’s recommendation of making a Master’s degree the entry level standard for dietetics practice (Hooker et al., 2012; Kicklighter, Cluskey, Hunter, Nyland, & Spear, 2013). This reinforces the need for a comprehensive curriculum designed to satisfy both theory and practice requirements for dietitian graduates. Although there is this extensive data available for the supply side of the dietetics profession, there seemed to be a lack of specific input from the demand perspective of potential employers of RDs and DTRs. It would most likely be of benefit to know: What knowledge and skills are characterized as essential or desirable for a successful job candidate? To this end, a study of position announcements for nutrition and dietetics jobs was undertaken.

## 2. Methods

From 2010 through 2013, we collected the information contained in two publicly available e-mail lists which post position announcements for jobs in nutrition and dietetics. These were: the New York State Registered Dietitian Yahoo group, and the Hunter College Nutrition and Food Sciences student and alumni listserv (NFS-L). Information from the New York Registered Dietitian Yahoo news group listings had been derived by searching the following websites (http://www.indeed.com/q-dietitian-l-New-York-state-jobs.html, https://www.linkedin.com, http://www.nutritionjobs.com, http://www.idealist.org, http://jobsearch.nytimes.monster.com/jobs, and http://eatrightny.org), using the key words: Dietitian, Dietician, Nutrition, and Nutritionist, singly or in combination. Positions posted to the NFS-L had been sent to Hunter Nutrition faculty for distribution by various area employers. In total, four hundred and twelve (412) individual job postings in the dietetics/nutrition field were recorded. Each job posting was assigned a unique identification code number. Duplicate postings were removed. These documents were then subjected to a content analysis as has been described elsewhere (Busch et al., 2012; Kris-Etherton et al., 2001). To conduct the content analysis of these postings, keywords were identified using the job categories defined by the Academy of Nutrition and Dietetics (AND) compensation and benefits survey (Rogers, 2014). This served as a starting point to enumerate the types of positions that have been posted for the New York metropolitan area in recent years.

### 2.1 Variable Extraction

The information gathered was categorized into the following variables:

- **Titles advertised:** Information was gathered on the exact job titles representing the advertised job positions.
- **Title categories:** The actual job position titles advertised were combined to form broader categories of 12 groups including: Registered Dietitian staff, administrative/management, community work-not RD, RD specialist, intern/volunteer, admin-not RD, research work, culinary, diet technician, sales, writer/media work and others. This was done to facilitate analysis and interpretation.
- **Complete name of the hiring organization:** The complete name of the hiring organization was recorded.
- **Employment sector:** Information was gathered on the types of industries requiring dietitians and nutritionists listed in the job postings. They included: non-profit organizations, for-profit organizations, government agencies, academia, and hospitals.
- **Advancement opportunities:** Information regarding advancement opportunities including the ability of the organizations to encourage professional growth was recorded.
- **Location:** The specific locations for the positions were recorded.
- **Responsibility level:** Job postings were categorized into several responsibility levels including: staff, supervisor, manager, director, executive, faculty/research, consultant, intern, volunteer and unknown.
- **On-site responsibilities:** information was gathered on the required work responsibilities listed for each job posting.
- **Work setting:** The types of facilities seeking employees were recorded.
- **Comorbid condition of client population:** Information was gathered on the comorbidities experienced by the patient populations requiring dietitian/nutritionist services.
- **Practice area of primary position:** The actual job posting titles were classified into practice areas as designated by the Academy of Nutrition and Dietetics (AND).
- **Academic degree qualification:** Academic qualifications including: Associates, Bachelors, Masters and Doctoral degrees required for each position were noted.
- **Credentials:** The credentials that candidates were required to possess including: RD-Registered Dietitian, DTR-Dietetic Technician Registered, CDE- Certified Diabetes Educator, Certified Dietitian-Nutritionist, Certified Nutrition Support Clinician, Certified Specialist in Pediatric Nutrition, Certified Dietary Manager and Certified Food Protection Professional were recorded.
- **Language:** Language requirements including: Spanish, French, Cantonese and Mandarin listed in the job postings were recorded. Information on additional language requirements including bi-lingual preference was also noted.
- **Knowledge and skills:** Information was gathered on the required and preferred knowledge and skills for each of the job postings.
- **Prior experience:** Information was gathered on the required number of years of working experience for each of the job postings.
- **Compensation:** Information regarding wages, salary and other forms of compensation were recorded for the job postings where available.
- **Work schedule:** Information on the required number of working hours daily including: full time, part time, fee for service/consulting or commensurate and the working time schedule including: weekdays, weekends and evenings were recorded.
- **Submit application to/at:** Information regarding the job search engines where the job postings were advertised and the full names of the human resource personnel designated to receive the job applications were documented.
- **Other:** Information regarding the job application websites and contact information including phone numbers, and email address were also recorded.

## 3. Content Analysis

A site license was obtained to utilize the *NVivo10*, a qualitative analysis software package (QSR International 2014) to extract categorization data from the raw position announcements. Through this content analysis, additional employer-desired skill and credential - specific categories were created. Content data extraction and coding were done by a graduate assistant (A.S.). Once the various data categories were created, quantitative statistical analysis was conducted using SAS version 9.0. Descriptive tests were carried out for the various categorical variables and results were reported as frequency distributions.

## 4. Results

**Figure 1:**
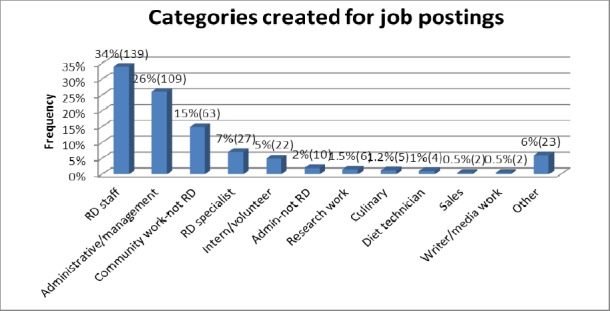
Categories created for job postings based on the Academy for Nutrition and Dietetics (AND)

Four hundred and twelve (412) individual job postings were recorded from the original data sources. The actual job posting titles advertised were condensed into broader categories of 12 groups to facilitate analysis and interpretation. Figure 1 shows that Registered Dietitian staff positions (RD staff) (34%, n=139); administrative/management positions (26%, n=109); community work positions (15%, n=63); Registered Dietitian specialists (7%, n=27); and intern/volunteer positions (5%, n=22); were the most frequently advertised job posting categories.

Job postings under the Registered Dietitian staff category were frequently advertised using the actual titles: Registered Dietitians (11%, n=46), dietitians (7%, n=27), and clinical dietitians (6%, n=24). Administrative/management category frequently included the job titles: clinical nutrition manager (2%, n=10), food service director (1.2%, n=5) and site coordinator (0.5%, n=2). The community work category included the job titles: nutritionist (5%, n=21), community nutrition educator (1%, n=4), and WIC nutritionist (0.7%, n=3). The Registered Dietitian specialist category included the job titles: Certified Diabetes Educator (1.2%, n=5), diabetes educator (0.5%, n=2), and practice diabetes educator (0.5%, n=2).

Job postings were further categorized by responsibility level and included: Executive, director, manager, supervisor, consultant, faculty/research, interns, and volunteers. Staff positions (65%, n=267), manager positions (14%, n=58), and director positions (9%, n=36) were the most frequently advertised (Figure 2a).

**Figure 2a:**
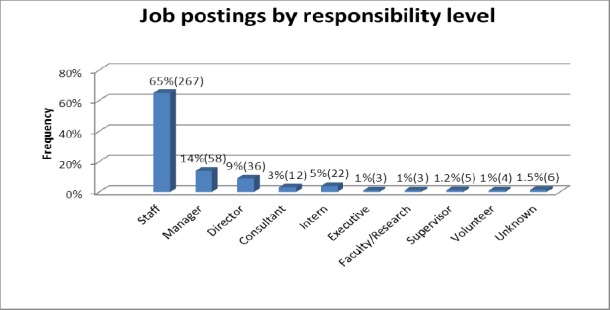
Distribution of advertised positions by responsibility levels

The most common types of facilities requiring dietitians/nutritionists included: acute inpatient hospitals, AIH (12%, n=51), food bank or assistance programs (5%, n=20), and food companies (4.6%, n=19) (Figure 2b). The diagnostic groups most frequently mentioned as patient populations requiring dietitian/nutritionist services were: HIV/AIDS (7%, n=29), diabetes (8%, n=32), obesity (5%, n=22), and eating disorders (2%, n=9).

**Figure 2b:**
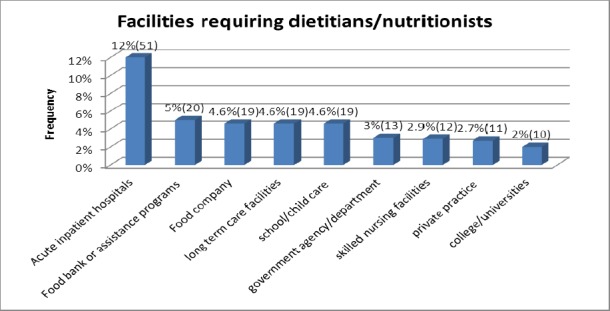
Facilities requiring dietitians/nutritionists

Consistent with the types of positions selected for this study, the majority of the job postings advertised required that candidates possess at least one of the following credentials: Registered Dietitian (RD) (33%, n=136), Certified Dietitian-Nutritionist (CDN) (5%, n=19), and Certified Diabetes Educator (CDE) (4%, n=18). Academic qualifications most frequently required were Bachelor’s degrees (50%, n=205) and Master’s degrees (28%, n=116) degrees, with only 2% requiring a doctoral degree. For those mentioning previous work experience, nine percent (n=38) of the employment opportunities required candidates to have obtained at most 5 years of relevant working experience.

**Figure 3:**
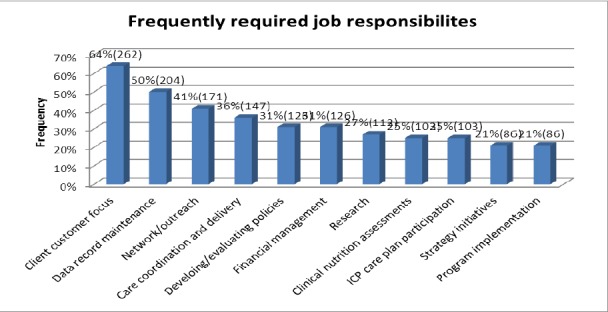
Frequently required job responsibilities among employment opportunities advertise

In terms of skills desired for these positions, client/customer focus (64%, n=262), data record maintenance (50%, n=204), and network/outreach skills (41%, n=171), were the most frequently listed in the job postings (Figure 3).

Additional skills and expertise required from candidates included: being able to communicate effectively (39%, n=162), having computer/Microsoft office skills (30%, n=122), flexibility to travel (24%, n=98), being detail oriented (20%, n=83), having clinical experience (17%, n=68), organizational skills (14%, n=59), research skills (15%, n=60), and fluency in Spanish (16%, n=57). Six percent (n=25) of job postings required candidates who were bilingual.

### 4.1 Interrelations between Job Categories and the Associated Job Responsibilities

Staff positions (65%, n=267), manager positions (14%, n=58), and director positions (9%, n=36), were the most commonly advertised job postings, based on responsibility level. (Figure 2a)

Registered Dietitian staff (48%, n=129), community work-not RD (21%, n=55), administrative/management (10%, n=26), and Registered Dietitian specialist (10%, n=26), were some of the common job title categories for the advertised staff positions (Figure 1). Actual job titles utilized in advertising the various staff positions commonly included: Registered Dietitian (16%, n=44), clinical dietitian (8.6%, n=23), dietician (9%, n=25), and nutrition technical advisor (6%, n=16). Staff positions usually required one year of experience. Having a bachelor’s degree was the most common academic qualification required (43%, n=114), and in few cases preferred (2%, n=6), for staff positions. Seven percent, (n=20) of the advertised staff positions required a Master’s degree, while 18% (n=49) positions preferred this. Almost half of the staff positions required that candidates possess a Registered Dietitian credential (42%, n=113), 6% (n=16) required a Certified Dietitian-Nutritionist credential (CDN), while 4% (n=11) preferred a Certified Diabetes Educator (CDE).

The most frequently required knowledge, skills and expertise for the staff positions included: effective communication (42%, n=112), proficiency in computer/Microsoft office (29%, n=77), detail oriented (18%, n=47), organizational (15%, n=40), and research skills (12%, n=33). Fluency in Spanish was required by 11% (n=29), and preferred by 7% (n=20), of the advertised staff positions, while 4% (n=12) preferred bilingual candidates.

Acute Inpatient Hospitals (AIH) were the most common types of facilities filling staff positions (13%, n=36). Others included: food bank or assistance program (5%, n=13), long term care (5%, n=13), and school/childcare (4%, n=10). client/customer focus (62%, n=166), data record maintenance (48%, n=128), and networking/outreach (42%, n=112) were the most commonly listed job responsibilities for the advertised staff positions. The patient populations which frequently required the services of the advertised staff positions included: HIV/AIDS (8%, n=22), diabetes (10%, n=27), and obesity (5%, n=12).

Manager positions were the second most commonly advertised job postings, based on responsibility level (14%, n=58) (Figure 2a). Administrative/management (72%, n=42) and community work-not RD (3%, n=2) were some of the common job title categories for the advertised manager positions (Figure 1). Actual job titles utilized in advertising the various manager positions commonly included: clinical nutrition manager (17%, n=10). Others included: health marketing manager, medical case manager, and diabetes care manager (2%, n=1).

Manager positions mostly required one to three years of work experience (14%, n=8), as well as a Registered Dietitian credential (14%, n=8). Having a bachelor’s degree was the most common academic qualification required (48%, n=28), while a Master’s degree required in 12% (n=7), and preferred in 16% (n=9) of all job postings for manager positions.

The frequently required knowledge, skills and expertise listed for manager positions included: effective communication (57%, n=33), detail oriented (38%, n=22), clinical experience (19%, n=11), proficiency in computer/Microsoft office (38%, n=22), and research (22%, n=13). Fluency in Spanish was required by 12% (n=7), and preferred by 11% (n=6) of all the related job postings. Acute inpatient hospitals (17%, n=10), food companies (14%, n=8) and school/childcare (10%, n=6) were the most common facilities seeking candidates for management positions. Client customer focus (62%, n=36), data record maintenance (52%, n=30), networking/outreach (39%, n=22), and financial management (35%, n=20) were some of the common work responsibilities listed for manager positions.

Director positions were the third most commonly advertised job postings, based on responsibility level (9%, n=36) (Figure 2a). Administrative/management (97%, n=35), and Admin-not RD (3%, n=1) were some of the common job title categories for the advertised director positions (Figure 1). Actual job titles utilized in advertising the various director positions commonly included: food service director (14%, n=5), and director of dinning services (6%, n=2).

Director positions frequently required one to three years of work experience (11%, n=4), and less commonly required a Registered Dietitian credential (8%, n=3). Half of the advertised director positions required a bachelor’s degree (50%, n=18), while 36% (n=13) required a Master’s degree as the minimum academic qualification. The frequently required knowledge, skills and expertise listed for director positions included: effective communication (31%, n =11), computer/Microsoft office (25%, n=9), food service (17%, n=6), management (14%, n=5), and organizational skills (12%, n=4).

Acute inpatient hospitals (8%, n=3), college/campus food (8%, n=3), and food bank or assistance program (6%, n=2), were the most common facilities posting director positions. Client/customer focus (69%, n=25), data record maintenance (61%, n=22), care coordination and delivery (44%, n=16), networking/outreach (39%, n=14), and financial management (28%, n=10) were some of the common work responsibilities listed for director positions.

## 5. Discussion

The descriptive statistics above can be compared to what has been previously said regarding needs for qualifications and skills in the dietetics workforce. The Registered Dietitian credential was the most common credential requirement listed in the job postings in our study (33%, n=136). This finding was consistent with results of the Compensation and Benefits Survey of the dietetics profession 2013, showing that Registered Dietitians had the ‘highest prevalence of dietetics related employment’ (84%, n=7,783) (Kris-Etherton et al., 2001). This same survey indicated that Dietetic Technician, Registered (DTR), had the second highest ‘prevalence of dietetics related employment’ (76%, n=1142) (Kris-Etherton et al., 2001). Our study recorded only 2% (n=5) of staff positions requiring this credential.

The Compensation and Benefits Survey of 2013 reported that almost half (47%) of the practicing Registered Dietitians had obtained Master’s degrees as their highest level of education. However, less than one-third of the job postings in our study listed a Master’s degree as a requirement (28%, n=116). These findings were not consistent with AND recommendations that a Master’s degree should serve as the entry level standard for dietetics practice (Hooker et al., 2012; Kicklighter et al., 2013). The Commission on Dietetic Registration (CDR) has announced that candidates must possess a Master’s degree in order to take the Registered Dietitian Examination as of 2024. Thus, whether they have specified so or not in their position announcements, any that require an RD, will *de facto* also require a Master’s degree.

### 5.1 Patient Populations

“The population of the United States continues to grow in racial and ethnic diversity, but RDs remain overwhelmingly white and female” (Hooker et al., 2012). Measurement issues (e.g. changing response categories and differing populations) preclude strict comparisons, but the AND Compensation Surveys show essentially no change from 2002 to 2011 in the percentage of RDs who are men (~3%), Asian (~5%), black (~2%), and Hispanic (~3%). This is true not only for RDs as a whole, but also for the most recent registrants (first 5 years), a group where increasing diversity might especially be expected.” This continues to be an area of concern for the dietetics profession and was reflected in our study results. We also found the need for language skills to be evident in our study results, with Spanish (16%), French (3%) and Cantonese (3%) being most often sought. While learning a second language is not routinely included in dietetics curricula, it is likely to enhance the versatility and hence employability of graduates. This finding is also reflective of a need for recruitment and training of more diverse dietitians and nutritionists, especially those with language skills.

### 5.2 Educational Needs and Standards Development

In the context of curriculum planning and development of future educational standards, we compared the results of this study to ACEND 2012 learning objectives for DPD and DI programs (ACEND, 2015), and identified how well these curriculum requirements matched up with identified workforce demands (Table 2).

**Table 1:** Interrelations between job categories and the associated job responsibilities

	Staff Frequency (N, %)	Manager Frequency (N, %)	Director Frequency (N, %)
**Academic qualification/credentials required**			
Bachelor’s degree	43% (114)	48% (28)	50% (18)
Master’s degree	7% (20)	12% (7)	36% (13)
Registered Dietitian credential	42% (113)	14% (8)	8% (3)
Certified Dietitian-Nutritionist (CDN)	6% (16)		
Certified Diabetes Educator (CDE)	4% (11)		
**Knowledge, skills and expertise required**			
Effective communication	42% (112)	57% (33)	31% (11)
Proficiency in MS Office	29% (77)	38% (22)	25% (9)
Detail oriented	18% (47)	38% (22)	
Organizational skills	15% (40)		
Research	12% (33)		
Fluency in Spanish	11% (29)	12% (7)	
**Common facilities requiring candidates**			
Acute Inpatient Hospitals (AIH)	13% (36)	17% (10)	8% (3)
Food bank or assistance program	5% (13)	14% (8)	6% (2)
School/childcare	4% (10)	10% (6)	
**Job responsibilities**			
Client / customer focus	62% (166)	62% (36)	69% (25)
Data record maintenance	48% (128)	52% (30)	61% (22)
Networking / outreach	42% (112)	39% (22)	39% (14)

**Table 2:** Skills prioritized by employers compared with ACEND 2012 learning objectives

Skills Identified	DPD Learning Objectives (9)	DI Learning Objectives (10)
Client customer focus	Suggestion to be incorporated in the DPD curriculum	Suggestion to be incorporated in the DI curriculum
Clinical Nutrition Assessments	**KRD 3.1** The curriculum should incorporate Medical nutrition therapy, development and implementation of nutrition interventions.	**CRD 3.1** Perform the Nutrition Care Process (a through e below) and use standardized nutrition language for individuals, and diverse population groups in a variety of settings
Medical Nutrition therapy		a. Assess the nutritional status of individuals, groups and populations in a variety of settings where nutrition care is or can be delivered
Data record maintenance		b. Diagnose nutrition problems and create problem, etiology, signs and symptoms (PES) statements
Maintain medical charts		c. Plan and implement nutrition interventions to include prioritizing the nutrition diagnosis, formulating a nutrition prescription, establishing goals and selecting and managing intervention
Care coordination and delivery		d. Monitor and evaluate problems, etiologies, signs, symptoms and the impact of interventions on the nutrition diagnosis
		e. Complete documentation that follows professional guidelines required by health care systems and guidelines required by the practice setting.
Financial Management	**KRD 4.5** The curriculum must include content related to coding and billing of dietetics/nutrition services to obtain reimbursement for services from public or private insurer	**CRD 4.6** Analyze quality, financial or productivity data and develop a plan for reimbursement **CRD 4.8** Ascertaining feasibility and cost effectiveness of services with consideration of costs and benefits. **CRD 4.9** Analyze financial data to assess utilization of resources **CRD 4.10** Develop a plan to provide or develop a product, program or service that includes a budget, staffing needs, equipment and supplies **CRD 4.11** Code and bill for dietetic/nutrition services to obtain reimbursement from public or private insurers.
Develop/Evaluates policies and procedures	**KRD 4.1** The curriculum should incorporate management training knowledge **KRD 4.2** The curriculum must include content related to quality management of food and nutrition services.	**CRD 2.8** Develop and utilize appropriate leadership skills **CRD 4.7** Utilize appropriate procedures to maximize use of available resources
ICP/CCP/Care plan participation	**KRD 3.1** The curriculum should incorporate Medical nutrition therapy, development and implementation of nutrition interventions	**CRD 2.5** Demonstrate active participation, teamwork and contributions in group settings
Strategy initiatives Program Implementation Networking/outreach	**KRD 4.1** The curriculum should incorporate management training knowledge **KRD 4.2** The curriculum must include content related to quality management of food and nutrition services.	**CRD 1.1** Select indicators of program quality and/or customer service and measure achievement of objectives **CRD 2.8** Develop and utilize leadership skills
Research Obtaining/updating literature	**KRD 1.1** The curriculum must reflect the scientific basis of the dietetics profession and must include research methodology, interpretation of research literature and integration of research principles into evidence based practice.	**CRD 1.2** Utilize evidence-based guidelines from notable sources including the DHHS, AHRQ and Cochrane databases in nutrition practice **CRD 1.4** Evaluate emerging research for application in dietetics practice **CRD 1.5** Utilize empirical research methods and procedures
Quality Assurance/Adherence	**KRD 4.2** The curriculum must include content related to quality management of food and nutrition services.	**CRD 1.1** Select indicators of program quality and/or customer service and measure achievement of objectives **CRD 2.8** Develop and utilize appropriate leadership skills
Oversee/train staff	**KRD 4.1** The curriculum should incorporate management training knowledge **KRD 4.2** The curriculum must include content related to quality management of food and nutrition services.	**CRD 2.4** Utilize evidence-based methods to effect changes in behavior **CRD 2.8** Develop and utilize appropriate leadership skills **CRD 3.2** Demonstrate effective communications skills for clinical and customer services in a variety of formats. **CRD 4.1** Participate in management of human resources
Diabetes education	**KRD 2.1** The curriculum must include opportunities to develop a variety of communication skills sufficient for entry into pre-professional practice. **KRD 2.2** The curriculum should incorporate validated standard methods of counseling **KRD 3.3** The curriculum must include education and behavior change theories and techniques.	**CRD 2.4** Utilize evidence-based methods to effect changes in behavior
Performance appraisals	**KRD 2.1** The curriculum must include opportunities to develop a variety of communication skills sufficient for entry into pre-professional practice. **KRD 2.2** The curriculum should incorporate validated standard methods of counseling **KRD 3.3** The curriculum must include education and behavior change theories and techniques.	**CRD 2.4** Utilize evidence-based methods to effect changes in behavior **CRD 2.8** Develop and utilize appropriate leadership skills **CRD 3.2** Demonstrate effective communications skills for clinical and customer services in a variety of formats. **CRD 4.1** Participate in management of human resources
Product/recipe development	**KRD 5.1** Food systems should be incorporated in the curriculum. “Course content must include the principles of food science and food systems, techniques of food preparation and application to the development, modification and evaluation of recipes, menus and food products acceptable to diverse groups”.	**CRD 3.3** Develop and deliver products, programs or services that promote consumer health, wellness and lifestyle management **CRD 3.6** identify and develop recipes, formulas and menus for acceptability and affordability that accommodate the cultural diversity and health needs of various populations, groups and individuals.
Computer skills/Microsoft Office	**Needs to be incorporated**	**Needs to be incorporated**

The table above is an example of how the skills most often listed as required or desired for positions in nutrition and dietetics might be met with some of the learning objectives for DPD and DI programs set forth by ACEND. While there is some degree of alignment, it is not complete. While some areas such as MNT are well represented, others are less so, and some are lacking.

While “client/customer focus” is implied by some of the KRs and CRDs, it is not explicitly stated as an objective. Since it has been identified as required or desired by the majority of employers, approaching this concept in a more overt way in nutrition and dietetics curricula would be desirable. “Data record maintenance” is related to documentation, which is related to the Nutrition Care Process (NCP), but likely also includes informatics, coding, and other factors. Computer skills and specifically proficiency in using Microsoft Office were stated as required or desired for many positions. While most dietetics education programs make use of common software and use computers to carry out both didactic and field assignments, it bears noting that these skills cannot be considered a “given” for all students. Resources should be made available to learn and practice computer skills as needed during education and training, and competency evaluated.

The major limitation of this study is its specificity to one geographical area. While it is probable that similar results would be found for other urban areas, with similar demographics, other areas may have different employee needs. A larger study including wider and more representative geographical areas is needed to generalize findings to broader population areas. Another limitation is that the position listings examined were from sources targeted specifically to Registered Dietitians, and dietetics students. These may not be representative of all the positions available to people with degrees in nutrition. A broader search may have captured more DTR and non-RD nutritionist positions.

## 6. Conclusion

This study has begun the exploration of the skills and credentials desired by the employers of nutrition and dietetics graduates. This is a perspective that has been lacking in previous literature on this topic. While our findings have supported the continued need for many of the ACEND 2012 learning objectives, others were absent from the list of skills most highly prioritized by these employers. Furthermore, the emphasis on client/customer focus, and need computer literacy, though they may be considered implicit in the ACEND objectives, may better be instilled in students by a more overt course of instruction on these topics.

**Compliance with Ethical Standards**

This project involved analysis of publicly available documents, and not human or animal subjects and so it was determined to be exempt from review by the City University of New York IRB. The study was funded by an internal PSC-CUNY grant and there are no potential conflicts of interest identified.
